# Electrophysiological characteristics of right- and left-sided Mahaim accessory pathways: A single-center experience in China

**DOI:** 10.3389/fcvm.2022.1052465

**Published:** 2022-12-07

**Authors:** Mingxian Chen, Zhuo Wang, Songyun Wang, Zhenjiang Liu, Xuping Li, Hui Yang, Lin Hu, Zhihong Wu, Qiming Li, Shenghua Zhou

**Affiliations:** ^1^Department of Cardiology, The Second Xiangya Hospital of Central South University, Changsha, China; ^2^Department of Cardiology, Wuhan Renmin Hospital of Wuhan University, Wuhan, China

**Keywords:** Mahaim, accessory pathway, decremental, tachycardiac, right-sided, left-sided

## Abstract

**Background:**

Mahaim-type accessory pathways (MAPs) are rare accessory pathways (APs) with specific properties. They are mostly located in the right side of the heart but rarely exist on the left side.

**Objectives:**

This study aims to analyze the clinical and electrophysiological (EP) characteristics of both-sided MAPs.

**Methods:**

A total of 2,249 patients with AP from our center were enrolled between 1 January 2011 and 27 March 2022. During the EP study (EPS) 17 patients were diagnosed with MAPs (right-sided: *n* = 13, left-sided: *n* = 4) according to the properties of Mahaim fibers.

**Results:**

MAPs constitute 0.75% of all APs. Out of 1,553 patients with left-sided APs, four patients (0.26%) were diagnosed with Mahaim fiber-mediated tachycardia. Out of 696 patients with right-sided APs, 13 patients (1.9%) were diagnosed with Mahaim fiber. Most Mahaim fibers were located at the free wall of the tricuspid and mitral annuli. Seven patients of right-sided MAPs were of atriofasicular type, six patients had right-sided MAPs, and all of the patients with left-sided MAPs were of atrioventricular (AV) type. The M potential only was detected in long-length MAPs. Coexistence with other supraventricular tachycardias (SVTs) was also observed both in patients with right-sided and left-sided MAPs. All the patients underwent radiofrequency ablation successfully. Only one patient had tachycardia recurrence during a follow-up.

**Conclusion:**

Although MAPs are commonly located at right sides, left sites are not impossible. The M potential contributes to the improvement of the successful ablation.

## Introduction

Mahaim and Benatt first described the Mahaim fiber leading to wide QRS tachycardia (1). Mahaim fibers are uncommon accessory pathways (APs) with specific characteristics. Mahaim-type accessory pathways (MAPs) usually originate from the atrial tissue, cross the atrioventricular (AV) junctions, and finally, insert into the branch of the His bundle (HB) (2). Therefore, MAPs are displayed as anterograde decremental conduction (AV-node-like) and a lack of retrograde conduction. MAPs are commonly located in the right-sided lateral region of the tricuspid annulus (3, 4). However, paraseptal and left-sided MAPs are rarely reported (5–7). Herein, we report on seven patients with right-sided MAPs and four patients with left-sided MAPs. The aim of this study is to describe the electrocardiographic and electrophysiologic characteristics in 17 patients with Mahaim fibers and to compare these findings between the patients with right-sided Mahaim fibers and those with left-sided Mahaim fibers.

## Materials and methods

This study enrolled 17 patients with MAPs between 1 January 2011 and 27 March 2022. The various electrophysiological (EP) properties of Mahaim fiber were retrospectively analyzed. Informed and written consent was obtained from study subjects. The study was approved by the Ethics Committee of the Second Xiangya Hospital of Central South University.

### EP study

**Figure 1:**
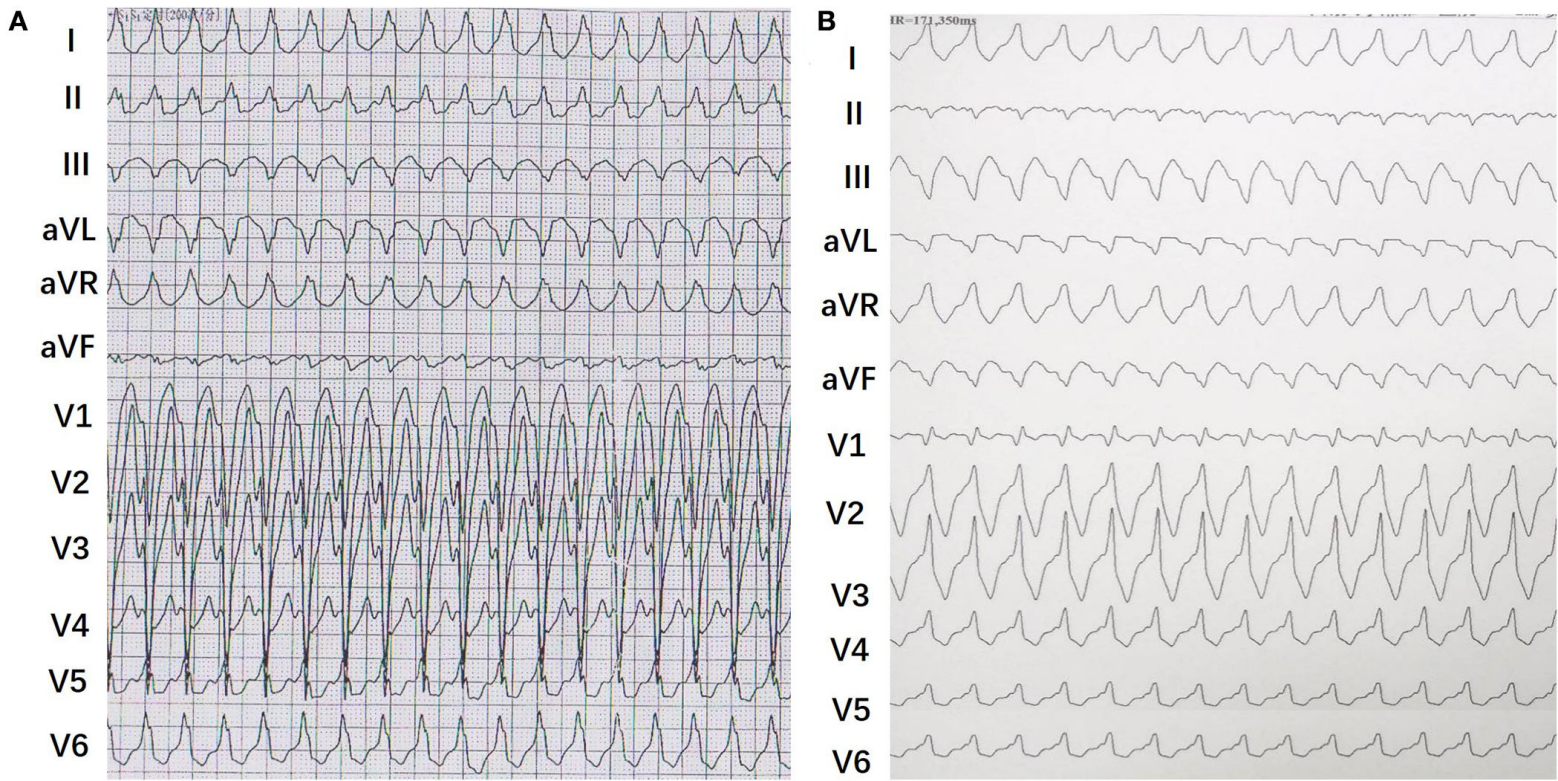
Representative 12-leads surface electrocardiograms of tachycardia in patients with right-sided and left-sided Mahain-type accessory pathways (MAPs). **(A)** It showed a typical wide QRS complex tachycardia with a left bundle branch block (LBBB) pattern. **(B)** It showed a typical wide QRS complex tachycardia with a right bundle branch block (RBBB) pattern.

**Figure 2:**
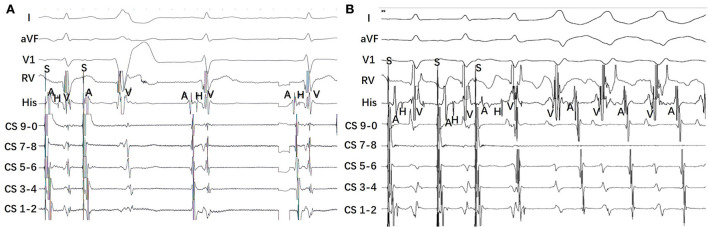
Representative intracardiac electrocardiogram from a patient with right-sided MAP and a patient with left-sided MAP. **(A)** Atrial programmed atrial stimulation (S1S1) induced a pre-excitation of QRS complex with LBBB pattern. It presents antegrade long conduction with lengthening of the A-H interval and A-V interval, a contemporary shortening of the H-V interval. **(B)** Atrial programmed stimulation (S1S1) induced tachycardiac with a broad QRS complex. The tachycardia has an RBBB pattern, long AV interval, and short VA interval. The earliest atrial activation was detected at the His-bundle electrodes during the tachycardia.

**Figure 3:**
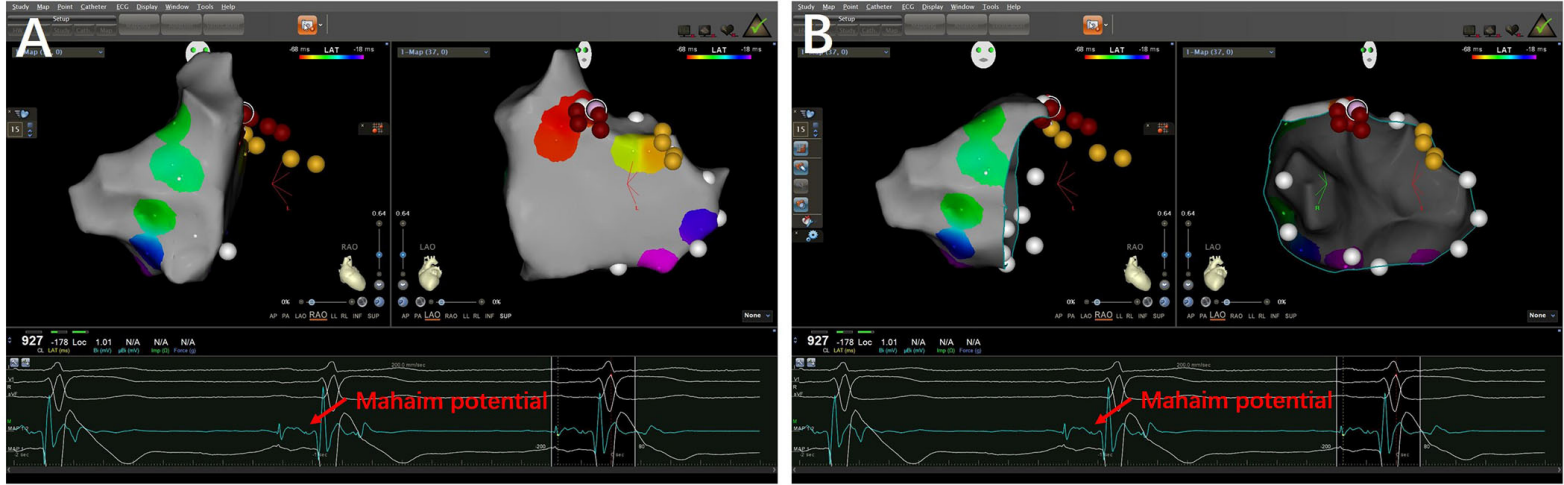
3-D mapping images of successful ablation sites in a patient with right-sided MAP. **(A)** The targets of Mahaim fiber located at the right anterior wall of tricuspid annulus (12:00 p.m.). **(B)** The target in ablated catheter presented Mahaim potentials.

During the study period, programmed electrical stimulation and recordings of the 12-lead surface ECG and intracardiac electrograms were performed by an electrophysiology system (Lead 2000; Sichuan Jinjiang Electronic Science and Technology Co., Ltd). The 3-D electroanatomic mapping and ablation were performed by CARTO system [Biosense Webster Co. or EP study (EPS); HT-Viewer system]. A Decapolar catheter was placed into the coronary sinus through the right internal jugular vein. Three quadripolar catheters were, respectively, positioned at the right ventricular (RV) apex, His bundle (HB), and right atrium. Programmed atrial stimulation and ventricular stimulation were performed to determine the antegrade conduction pattern to the ventricle and the retrograde conduction pattern to the atrium. For patients with right-sided MAP, atrial programmed stimulation induced tachycardia with broad complex LBBB surface ECG morphology. Left-sided MAP in patients induced tachycardia with broad complex RBBB surface ECG morphology (Figure 1). The M potential was recorded during sinus rhythm and tachycardia. The characteristics of Mahaim fibers were defined as follows: (1) baseline normal QRS without pre-excitation or minimal pre-excitation; (2) programmed atrial pacing inducing manifest pre-excitation and a progressive increase in AH and AV interval along with a shortening of HV interval (Figure 2); (3) absence of retrograde conduction over the AP during ventricular stimulation; and; (4) during antegrade pre-excitation, SVT retrograde His bundle activation was detected.

V_Abl_-QRS was defined as the interval between the beginning of ventricular ECG and the beginning of the QRS complex. The type of MAPs V_Abl_-QRS was determined by the V_Abl_-RV_Apex_ interval and was treated as the interval between the beginning of ventricular ECG at the successful ablation point and the ventricular ECG recorded from the RV apex. Vabl-RVapx intervals contribute to the guidance of MAPs ablation.

### RF ablation

After MAP was confirmed by the EP properties, a catheter (8 Fr, 3.5-mm-tip electrode, DD or JJ curve, 1-6-2 mm spacing, 115 insertion length, Thermocool SmartTouch SF^®^ Biosense Webster Co.; or EPS, HT-Viewer system) was used for mapping and ablation. The MAPs were ablated with a target contact force of 10–15 g, a target ablation index (AI) of 500–600 watts/grams/second, and an impedance drop of 5–10 Ω. The tricuspid annulus and mitral annulus were mapped during sinus rhythm. For the left-sided MAPs, the transseptal approach was performed. The M potential was detected between atrial and ventricular intracardiac potential during sinus rhythm (Figure 3). The AM interval (between the beginning of the atrial electrogram and the M potential) and MV interval (between the M potential and the beginning of the ventricular electrogram) were recorded. The M potential was recorded at the atrial insertion site of the Mahaim fibers. It was considered a good predictor of a suitable ablation site (8). Successful ablation includes no ventricular pre-excitation, no AP conduction, and no inducible tachycardia observed during repeated programmed stimulation.

### Statistical analysis

SPSS software (version 23.0; SPSS, Inc., Chicago, IL) was used for statistical analyses. The medians and minimum-maximum were presented for the non-normally distributed variables. The Mann-Whitney *U*-test was performed to compare the differences in non-normally distributed variables between the right-sided group and the left-sided group. Categorical variables were analyzed by the chi-square test. A *p*-value < 0.05 was considered statistically significant.

## Results

The records of 2,249 patients with AP who underwent EPS were evaluated. Among these patients, 1,553 (69.1%) were diagnosed with left-sided APs and 696 patients (30.9%) were diagnosed with right-sided APs. In our center, MAPs constituted 0.75% of all APs. Out of 1,553 patients with left-sided APs, four patients (0.26%) were diagnosed with Mahaim fiber-mediated pathway. Out of 696 patients with right-sided APs, 13 patients (1.9%) were diagnosed with MAPs. According to the abovementioned results, it was observed that these APs were more often right-sided rather than left-sided.

### Clinical characteristic analysis

The baseline characteristics of the 17 patients are summarized in Table 1. Approximately 54.8% were female in the right-sided group, while 50% were male in the left-sided group. The mean age was 47 (28–66) years in the right-sided group, while it was 56 (31–79) years in the left-sided group. All of the patients had palpitations, and none of them had syncope. Antiarrhythmic drugs were prescribed before RF ablation therapy for three patients in the right-sided group and two patients in the left-sided group. A resting 12-lead ECG revealed a minimal pre-excitation during sinus rhythm in five patients of the right-sided group and in one patient of the left-sided group.

**Table 1:** Clinical characteristics of patients with MAPs.

**Characteristic**	**Right side (*n* = 13)**	**Left side (*n* = 4)**	***P*-value**	***X* ^2^**
Female, *n* (%)	54.8% (7/13)	50% (2/4)	0.89	0.018
Age, years	47 (28-66)	56 (31-79)	0.42	
**Clinical features**				
Palpitation	100% (13/13)	100% (4/4)		
ECG documented tachycardia	84.6% (11/13)	100% (4/4)	0.40	0.697
Syncope	0% (0/13)	0% (0/4)		
**Drugs**				
Calcium channel blocker	23.1% (3/13)	25% (1/4)	0.87	0.023
Beta blocker	38.5% (5/13)	25% (1/4)	0.62	0.243
No medication	38.5% (5/13)	50% (2/4)	0.68	0.168
**Electrocardiogram**				
Minimal pre-excitation during sinus rhythm	38.5% (5/13)	25% (1/4)	0.62	0.243
No pre-excitation	61.5% (8/13)	75% (3/4)	0.62	0.243

### Electrophysiological study

Tables 2, 3 show the EPS characteristics of the patients studied. Atrial pacing protocol showed manifest pre-excitation with decremental AV conduction in all patients. With incremental atrial pacing, AH was prolonged, HV was shortened, and tachycardia was induced (Figure 2).

**Table 2:** Electrophysiological data from 17 patients with Mahaim-type accessory pathways who underwent electrophysiological study.

**Parameter**	**Right-sided**	**Left-sided**	***P*-value**	***X* ^2^**
**EPS**				
QRS duration	132 (111–159)	150 (133–169)	0.17	
Basic cycle length, ms	691 (540–813)	717 (613–825)	0.43	
M potential	53.9% (7/13)	0% (0/4)	0.06	3.662
SVT cycle length, ms	325 (242–379)	332 (299–369)	0.97	
**Combined other tachycardia**				
AVRT or concealed AP	15.4% (2/13)	25% (1/4)	0.66	0.195
AVNRT	30.7% (4/13)	25% (1/4)	0.78	0.17
AT	7.7% (1/13)	0	0.56	0.327
**Ebstein's disease**	0	0		
**Procedure**				
Procedure duration, min	111 (89–221)	82 (62–133)	0.17	
RF duration, min	11 (2–38)	3 (2–3)	0.03	
**Recurrence**	7.7% (1/13)	0% (0)	0.56	0.327

AP, accessory pathway; AT, atrial tachycardia; AVRT, atrioventricular reentrant tachycardia; AVNRT, atrioventricular nodal reentrant tachycardia; EPS, electrophysiological study; SVT, supraventricular tachycardia; RF, radiofrequency.

**Table 3:** EPS of all patients.

**No**	**Side of MAP**	**Combined arrhythmias**	**Minimal pre-excitation**	**M potential**	**AM**	**MV**	**V_abl_-QRS**	**V_abl_-RV_Apex_**	**AP type**	**AP length**
1	R1	AVNRT	+	+	76	33	−13	−22	AF	Long MAP
2	R2	None	—	—	None	None	9	49	AV	Short MAP
3	R3	AVNRT	+	+	83	69	−3	−13	AF	Long MAP
4	R4	None	+	+	114	27	−7	−26	AF	Long MAP
5	R5	None	—	—	None	None	6	33	AV	Short MAP
6	R6	None	—	—	None	None	9	51	AV	Short MAP
7	R7	AVNRT + Concealed AP	+	+	111	42	−11	−19	AF	Long MAP
8	R8	None	—	—	None	None	13	66	AV	Short MAP
9	R9	AVRT	—	+	87	44	−6	−21	AF	Long MAP
10	R10	None	—	—	None	None	14	49	AV	Short MAP
11	R11	AVNRT	+	+	43	61	−5	−9	AF	Long MAP
12	R12	None		—	None	None	10	54	AV	Short MAP
13	R13	AT		+	43	9	−8	−26	AF	Long MAP
14	L1	AVNRT + Concealed AP	—	—	None	None	11	49	AV	Short MAP
15	L2	None	—	—	None	None	22	69	AV	Short MAP
16	L3	None	+	—	None	None	26	53	AV	Short MAP
17	L4	None	—	—	None	None	33	62	AV	Short MAP

AF, atriofasicular; AP, accessory pathway; AM, the interval between the beginning of the atrial ECG and the M potential; AT, atrial tachycardia; AV, atrioventricular; AVRT, atrioventricular reentrant tachycardia; AVNRT, atrioventricular nodal reentrant tachycardia; EPS, electrophysiological study; L, left side; MV, the interval between the M potential and the beginning of ventricular ECG; MAP, Mahaim accessory pathway; SVT, supraventricular tachycardia; R, right side; RF, radiofrequency; V_Abl_-QRS, the interval between the beginning of ventricular ECG and the beginning of the QRS complex; V_Abl_-RV_Apex_ interval, the interval between the beginning of ventricular ECG at the successful ablation point and the ventricular ECG recorded from the RV apex.

For right-sided and left-sided MAPs, the median paced duration of the QRS interval was 132 ms (111–159) and 150 ms (133–169), respectively (*P* = 0.17). The median of basic cycle length was 691 ms in the right-sided and 717 ms in the left-sided groups, respectively (*P* = 0.43). Only seven patients with right-sided MAPs presented with “Mahaim potential,” but no patients with left-sided MAPs were observed to have M potentials. After ablation, all of the M potentials were eliminated. The median SVT cycle length was 325 ms in the right-sided and 332 ms in the left-sided groups, respectively (*P* = 0.97).

Among 11 patients with right-sided MAPs, there were two patients found with atrioventricular reentrant tachycardia (AVRT) or concealed AP, four patients accompanied with atrioventricular nodal reentrant tachycardia (AVNRT), and one patient accompanied with AT. In four patients with left-sided MAPs, only one patient was induced with AVRT or concealed AP and one patient was accompanied with AVNRT. None of the patients had Ebstein's disease. Radiofrequency ablation was delivered in temperature-controlled mode at a target temperature of 55–60°C and a power output of 30–35 W in all the cases. All of the patients underwent successful ablation, and the median procedure duration time was 111 min in the right side and 82 min in the left side groups, respectively (*P* = 0.17). The median RF ablation duration time was 11 min in the right side and 3 min in the left side groups, respectively (*P* = 0.03). Only one patient with right-sided MAPs had recurrence during the 1-year follow-up.

**Figure 4:**
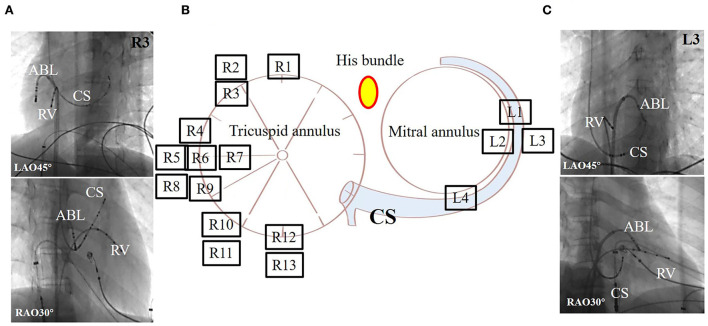
Representative fluoroscopic images of successful ablation sites in two patients with Mahaim-type accessory pathway of 17 patients. **(A)** The fluoroscopic images in a patient with right-sided MAP. **(B)** Solid circles show radiofrequency ablation targets around the tricuspid and mitral annuli. There were 13 patients showing MAPs around the tricuspid annulus, but for most patients they were located at the free wall. In total, four patients had them around the mitral annulus, but in most of the patients, it was located at the free wall. The dot was represented as his bundle. **(C)** The fluoroscopic images in a patient with left-sided MAP. ABL, ablation catheter; CS, coronary sinus; L, patient with left-sided Mahaim; LAO, left anterior oblique; R, patient with right-sided Mahaim; RAO, right anterior oblique; RV, right ventricular.

According to our study, MAPs were located on the tricuspid annuli (right-sided, *n* = 13) and mitral annuli (left-sided, *n* = 4). In the right-sided group, one patient had anteroseptal MAP, two patients with anterolateral MAP, six patients with lateral MAPs, two patients with posterolateral MAP, and two patients with posteroseptal MAP. In the left-sided group, there were three patients with anterolateral MAPs and one patient with posterolateral MAP (Figure 4).

## Discussion

This study evaluated clinical and EP characteristics in patients with right-sided and left-sided MAPs. The main findings were as follows: (1) the incidence of MAPs was very low in APs. MAPs mainly existed in the lateral aspect of the tricuspid annulus, but could also exist in the mitral annulus. (2) The success rate of catheter ablation was very high. (3) Not all of the right-sided MAPs have M potential, but we did not detect the M potential in the left-sided MAP. The M potential provided a suitable target for the ablation of MAPs and improved the successful ablation.

Mahaim-type accessory pathways are rare APs with unique properties. They were usually found in the right side of the heart. MAPs in the left side are very rare, and only a few cases have been reported. In our center, MAPs constitute 0.75% of all APs. Out of 1,553 patients with left-sided APs, four patients (0.26%) were diagnosed with Mahaim fiber-mediated pathway. MAPs in right-sided patients are more common than in left-sided. Consistent with a previous study (9), MAPs were commonly located at the lateral tricuspid annulus site (10–12). However, the incidence in our study is much lower than others (9). This may be due to regional differences between different countries. Ozcan et al. indicated that it was possibly ascribed to the fact that their center is a referral hospital for atypical AP ablation. Incidence may vary from region to region or race to race. Therefore, it is a more representative response to the prevalence of MAPs.

Yanni et al. demonstrated that the “primary ring” formed the AV node and the AV conduction axis which only contributed to the tricuspid annulus. MAPs originated from inferior extensions of the AV node. Therefore, right-sided MAPs are more commonly found than left-sided MAPs. MAPs are AV ring tissues, which are the remnants of the primary ring and have the similar properties to AV node and Kent bundle (13). Therefore, MAPs display AV node-like conduction properties and a slight pre-excitation. Recently, more cases with left-sided MAPs were reported (9). It was hypothesized that left-sided MAPs were attributed to a defect in embryological migration accompanied by AV isolation defect.

**Figure 5:**
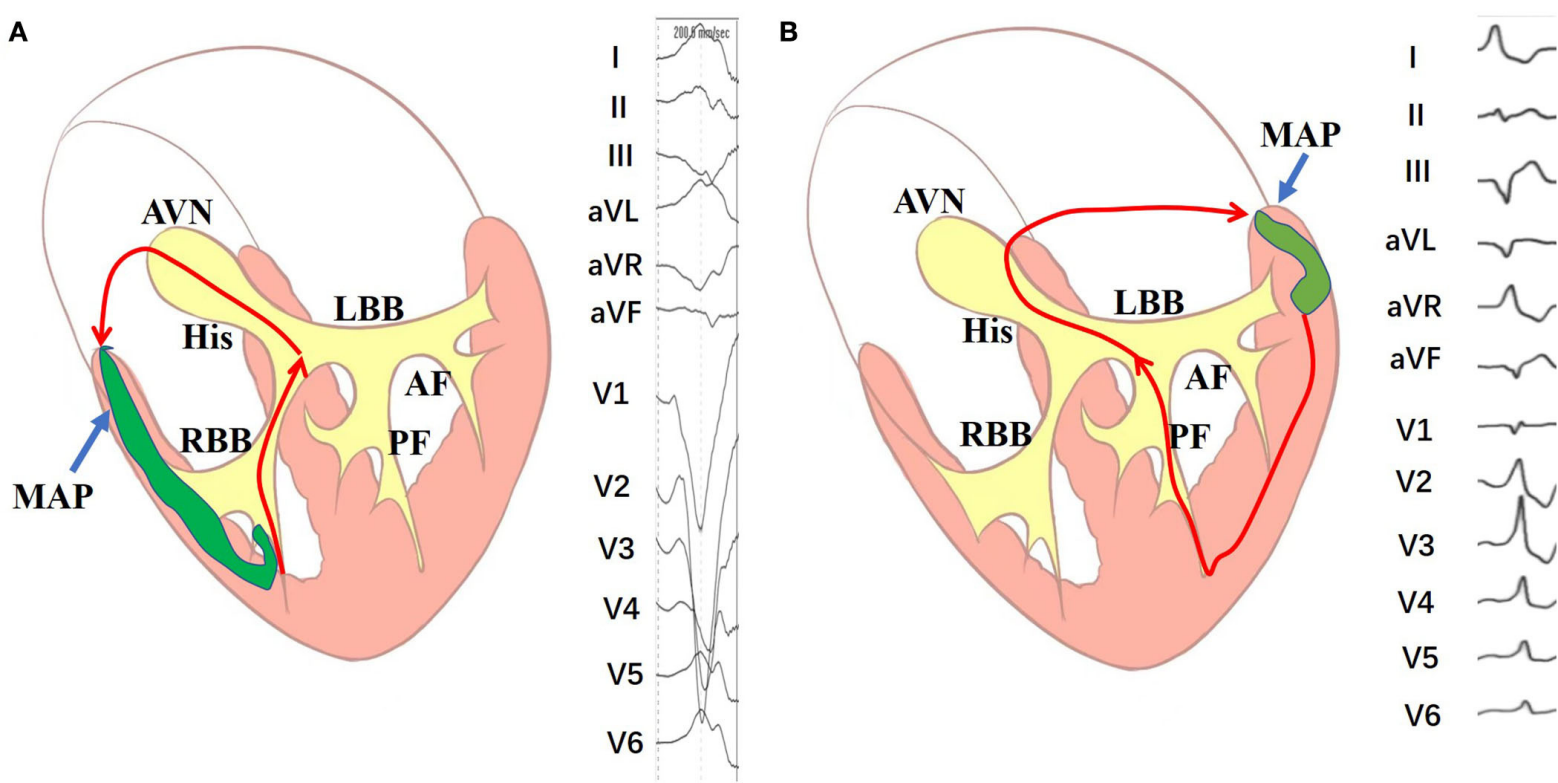
Schematic diagram of the mechanism of the long-length and short-length Mahaim-mediated tachycardias. **(A)** The mechanism of right-sided long-length Mahaim-mediated tachycardias. **(B)** The mechanism of left-sided short-length Mahaim-mediated tachycardias. AF, anterior fascicle; AVN, atrioventricular node; LBB, left bundle branch; MAP, Mahaim accessory pathway; PF, posterior fascicle; RBB, right bundle branch.

The length of the MAPs was determined by Vabl-QRS intervals. If the earliest ventricular potentials in the ablated MAPs were later at QRS complexes (a negative Vabl-QRS interval), it was considered long-length MAPs and defined as atriofasicular MAPs (Table 3). It was a long accessory from atrial insertion to ventricular insertion. If the earliest ventricular potentials in the ablated MAPs were preceded the surface QRS complexes (a positive Vabl-QRS interval), it was considered short-length MAPs and defined as AV MAPs (Figure 5). In those cases, all of the left-sided MAPs and parts of the right-sided MAPs were short-length MAPs and AV MAPs. Long-length MAPs were only existed in the right side. It indicated that long-length MAPs were just only appeared in the right-sided. However, the left-sided MAPs just only presented as short-length MAPs. Short-length MAPs also existed in the right side. In our cases, the M potential was not detected in all patients. We found that parts of patients with right-sided MAPs and all of the patients with left-sided MAPs were not observed M potential. M potential were only detected in long-length MAPs or atriofasicular MAPs. The M potential is helpful for the improvement of successful ablation. The earliest ventricular potential as well as Vabl-RVapx intervals contribute to the guidance of MAPs ablation in patients without M potentials.

Both the right-sided and left-sided MAPs have similar clinical and EP characteristics. In the present study, we found that the AM and the MV intervals were longer in the right-sided MAPs than that in the left-sided MAPs. Ozcan et al. demonstrated that they were associated with decremented conduction rather than the length of the MAPs. Previous studies have reported that the coexistence of AVNRT in patients with right-sided MAPs was from 9 to 40% (9, 14–16). However, in our center experience, we found that the incidence of AVNRT was 42.8% and the incidence of AVRT was 28.3%. Ozcan et al. found that no AVNRT coexisted in patients with left-sided MAPs (9). However, in our study, we found that one patient was with AVNRT and one patient was with AVRT. It indicated that the coexistence was not specific to the right-sided MAPs but also existed in the left-sided MAPs.

### Limitations

The present study included only 17 patients with MAPs, which is a very small number for an observational, retrospective, and single-center study. More patients with MAPs in other EP centers could be involved in future studies.

## Conclusion

The reported incidence of MAPs is very low. MAPs are generally located in the right side and occasionally the left. The M potential only is detected in long-length MAPs. The coexistence of other arrhythmias is also a common phenomenon. The radiofrequency ablation of MAPs has high success rates.

## Data availability statement

The raw data supporting the conclusions of this article will be made available by the authors, without undue reservation.

## Ethics statement

The studies involving human participants were reviewed and approved by the Second Xiangya Hospital of Central South University. The patients/participants provided their written informed consent to participate in this study. Written informed consent was obtained from the individual(s) for the publication of any potentially identifiable images or data included in this article.

## Author contributions

MC, ZWa, and SZ participated in the study design and drafted the manuscript. HY, ZL, XL, ZWu, SW, and LH contributes to data collection. MC and ZWu were responsible for writing the manuscript. HY, ZL, XL, and QL contributes to the manuscript revision. All authors contributed to the article and approved the submitted version.
